# Adsorption and Fenton-like Degradation of Ciprofloxacin Using Corncob Biochar-Based Magnetic Iron–Copper Bimetallic Nanomaterial in Aqueous Solutions

**DOI:** 10.3390/nano12040579

**Published:** 2022-02-09

**Authors:** Hongrun Liu, Yuankun Liu, Xing Li, Xiaoying Zheng, Xiaoying Feng, Aixin Yu

**Affiliations:** Municipal Engineering Department, College of Civil Engineering and Architecture, Beijing University of Technology, Beijing 100124, China; liuhongrun7768@163.com (H.L.); lixing@bjut.edu.cn (X.L.); zhengxiaoying@bjut.edu.cn (X.Z.); 18801365714@emails.bjut.edu.cn (X.F.); yuaixin809@163.com (A.Y.)

**Keywords:** fenton-like catalyst, corncob biochar-based magnetic iron–copper bimetallic nanomaterial, ciprofloxacin, adsorption, advanced oxidation process, AOPs, catalytic activity

## Abstract

An economical corncob biochar-based magnetic iron–copper bimetallic nanomaterial (marked as MBC) was successfully synthesized and optimized through a co-precipitation and pyrolysis method. It was successfully used to activate H_2_O_2_ to remove ciprofloxacin (CIP) from aqueous solutions. This material had high catalytic activity and structural stability. Additionally, it had good magnetic properties, which can be easily separated from solutions. In MBC/H_2_O_2_, the removal efficiency of CIP was 93.6% within 360 min at optimal reaction conditions. The conversion of total organic carbon (TOC) reached 51.0% under the same situation. The desorption experiments concluded that adsorption and catalytic oxidation accounted for 34% and 66% on the removal efficiency of CIP, respectively. The influences of several reaction parameters were systematically evaluated on the catalytic activity of MBC. OH was proved to play a significant role in the removal of CIP through electron paramagnetic resonance (EPR) analysis and a free radical quenching experiment. Additionally, such outstanding removal efficiency can be attributed to the excellent electronic conductivity of MBC, as well as the redox cycle reaction between iron and copper ions, which achieved the continuous generation of hydroxyl radicals. Integrating HPLC-MS, ion chromatography and density functional theory (DFT) calculation results, and possible degradation of the pathways of the removal of CIP were also thoroughly discussed. These results provided a theoretical basis and technical support for the removal of CIP in water.

## 1. Introduction

The environmental deterioration by the excessive discharge of pollutants has aroused great concern all over the world [1]. In particular, personal care products (PPCPs), such as antibiotics, medicines, cosmetics, and fungicides, have recently posed a serious threat to the aquatic environment [2,3]. Antibiotics are the most widely used among them in medicine, animal disease prevention, aquaculture, and animal husbandry [4]. It has been reported that antibiotics have a severe impact on the environment and human health, although in small concentrations, even at trace levels [5,6]. Moreover, antibiotics have been detected in the water on a global scale with increasing use [7].

Ciprofloxacin (CIP), as the third generation of quinolones, is one of the most common antibiotics. Because of its chemical stability and antimicrobial activity, it is widely used and detected in the aquatic environment [8]. The primary sources of CIP pollution include domestic discharges, hospital effluent, and wastewater from the pharmaceutical industry, which can cause damage to the ecosystem and the immune systems of consumers [9].

Due to the high ecological toxicity and difficult degradation of CIP, it cannot be effectively removed by traditional treatment methods from the aquatic environment [10,11,12,13]. In biological processes, antibiotics will inhibit the reproduction of microorganisms, and there is a risk of producing drug-resistant bacteria [14]. The characteristic of physical treatment is that pollutants cannot be completely degraded from the environment. Therefore, it is essential to develop an effective treatment method to avoid the above problems [15].

The advanced oxidation process (AOP) is characterized by mild reaction conditions, a high reaction rate, and a good effect on the decomposition of antibiotics; therefore, it has become the focus of research. In recent years, various published papers have been presented with some methods for the AOP, for example: Fenton oxidation, ozonation, photocatalytic oxidation, and electrochemical oxidation [16,17,18,19,20]. These methods can effectively degrade CIP. Among them, Fenton oxidation is considered, because the hydroxyl radical (·OH) produced by Fenton reaction has low selectivity and high reactivity, and can oxidize organic molecules until completely mineralized [20]. In general, transition metals such as Fe, Cu, Co, and Ni have been used in Fenton oxidation. Because iron has excellent magnetism and redox properties, it is widely used in various technical fields. However, the activity of iron oxide is low, thus it is the formation of bimetallic oxides by introducing a second metal compound [21]. More OH are produced in the catalytic oxidation reaction due to Cu and its oxides’ better redox properties [22]. Therefore, the iron and copper bimetallic composite synthesized can enhance catalyst activity. However, metals as catalysts are prone to agglomeration and metal ion leaching and cause secondary pollution. These problems can be overcome by loading iron–copper bimetal onto carbon-based [23]. In addition, the synergistic action between metal and carbon-based can significantly enhance the performance of H_2_O_2_ activation.

Biochar is a carbonaceous material produced by biomass pyrolysis with abundant raw materials. It is considered as a promising and low-cost adsorbent for organic pollutants [24]. Biochar has the characteristics of a loose and porous highly specific surface area, and abundant surface oxygen-containing functional groups, which gives biochar good adsorption performance [25]. According to the report from the United States Department of Agriculture, approximately 40–50 million metric tons of corncobs could be collected annually in the world. However, corncobs are mostly burned, resulting in a waste of resource [26]. Agricultural waste corncob is a by-product of corn processing and characterized by rich carbon and low ash content. Therefore, the magnetic bimetallic composite embedded into the corncob can enhance the recycling performance of materials and accelerate the electron transfer, thus promoting the activation of H_2_O_2_ [27,28].

In this work, an economical corncob biochar-based magnetic iron–copper bimetallic nanomaterial (marked as MBC) was synthesized through a co-precipitation and pyrolysis method. The best novelty of our work is that the loading and dispersion function of biochar can overcome the inherent aggregation of metals, and the carbon-based as a medium promoted the circulation of metal ions and enhanced the activation of hydrogen peroxide. Moreover, this work had the preparation of using waste biomass as catalysts to improve the degradation of organic pollutants from aqueous solutions to realize the utilization of waste resources and cost-saving, which was the significance of preparing MBC. MBC has suitable magnetic, and easy to recycle, adsorption and catalytic activity. The significance in practical treatment is easy separation in order to achieve reuse and avoid secondary pollution. MBC has good removal efficiency for pollutant and is feasible for the advanced treatment of sewage such as medical wastewater.

For this purpose, the optimal preparation scheme was determined to enhance the removal efficiency of CIP. Additionally, the characteristics of catalysts were analyzed by means of energy dispersive scanning electron microscope (SEM), X-ray diffraction (XRD), Fourier-transform infrared spectroscopy (FTIR), X-ray electron spectroscopy (XPS), and vibration sample magnetometer (VSM). Subsequently, the degradation properties under different systems and influencing factors were studied on the degradation of CIP. The relationship between adsorption and catalytic oxidation was analyzed in this study. Additionally, the stability of the catalyst was explored under different conditions. The possible catalytic mechanism was studied by the EPR and quenching reactions. Ultimately, the possible intermediates and degradation pathways were also investigated via HPLC-MS, IC and DFT calculation in this work.

## 2. Materials and Methods

### 2.1. Materials

Corncob powders (100 mesh) were bought from agricultural product processing centers in Shandong in China. All the chemicals were analytically pure, and ultrapure water was used to configure all solutions (Text S1).

### 2.2. Preparation of Catalysts and Characterization

Corncob powders were washed and dried naturally before use. Firstly, the prepared corncob powders were heated by a tube furnace to 500 °C for 2 h under N_2_ atmosphere. Secondly, the cooled product was ground and sifted (100 mesh). Finally, it was stored in a vacuum dryer and named BC.

**Figure 1:**
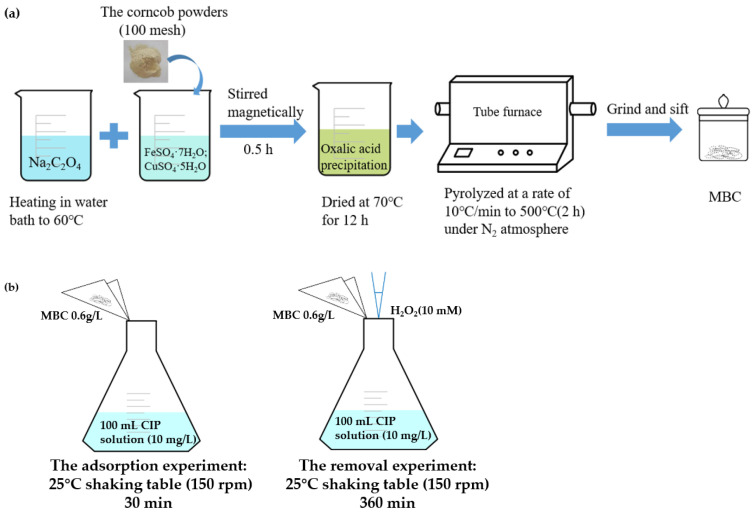
(**a**) The preparation route of MBC catalyst; (**b**) the schematic diagram of the experimental parameters.

As depicted in Figure 1a, corncob biochar-based magnetic iron–copper bimetallic nanomaterial (marked as MBC) was synthesized through chemical co-precipitation. Firstly, 50 mL 0.3 M Na_2_C_2_O_4_ was heated to 60 °C in a water bath. Secondly, a certain amount of corncob powders, FeSO_4_·7H_2_O, and CuSO_4_·5H_2_O were simultaneously added to 50 mL ultrapure water. Then, the mixed solution was quickly and evenly added to the prepared Na_2_C_2_O_4_ and magnetically stirred for 30 min. Finally, in order to remove the unreacted reactants, the obtained yellow-green oxalic acid precipitation was repeatedly centrifuged and washed several times. The resulting product was dried at 70 °C for 12 h. The pyrolysis steps for synthesizing MBC were the same as BC.

The iron and copper bimetallic complex (no corncob powders) were labeled as FeCu. Except for the second step without corncob, the other synthesis steps of FeCu were the same as MBC.

The characterization of catalysts was described in Text S2.

### 2.3. Experimental Section and Analytic Methods

The experiments of determining the optimal preparation scheme were conducted at 25 °C in a constant temperature shaking table (150 rpm) (SHA-CA, China) for 30 min (Figure 1b). The quality range of corncob powders was 1.0–5.0 g and the ratio of iron to copper remained unchanged (0.2 M FeSO_4_·7H_2_O and 0.1 M CuSO_4_·5H_2_O) to study the adsorption efficiency of CIP. Then, the different Fe/Cu molar ratios were 3:0, 3:1, 2:1, 1:1, 1:2 to choose the optimal material preparation conditions.

All the removal experiments were conducted at 25 °C in the shaking table (150 rpm) for 360 min (Figure 1b). The removal reaction of CIP was performed by adding the MBC or BC (0.6 g/L) together with H_2_O_2_ (10 mM) into 100 mL CIP solution (10 mg/L). A certain amount of solution was removed from the reactor at specified intervals and quenched with excess methanol. Comparison with the above experiments, experiments without H_2_O_2_, or catalysts were set under the same condition as the control. Subsequently, the batch experiments of influencing factors were performed, including initial pH, H_2_O_2_ concentration, catalyst dosage, initial CIP concentrations, and the organic and inorganic substances. A total of 1 M HCl or 0.1 M NaOH was used to adjust the pH of the CIP solution to explore the influence of pH.

The recycling experiments were prepared under four conditions (without washing, water washing, ethanol washing (95%), and hydrochloric acid washing (0.1 M HCl)). The quenching reactions were performed by adding chemical agents tert-Butanol (TBA) and potassium iodide (KI), respectively [29]. All the samples were filtered through a 0.45 μm membrane before being detected. The absorbance of ciprofloxacin solution was detected at 277 nm by the UV-Vis spectrophotometer (UV-1900, Shimadzu, Japan). The other analytic methods were described in Text S3. All the above experiments were repeated twice, and the average values were taken as the experimental results.

## 3. Results and Discussion

### 3.1. Characterization

#### 3.1.1. SEM and BET Analysis

**Figure 2:**
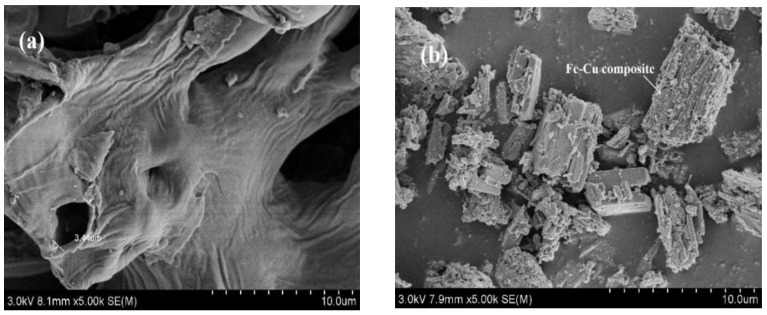

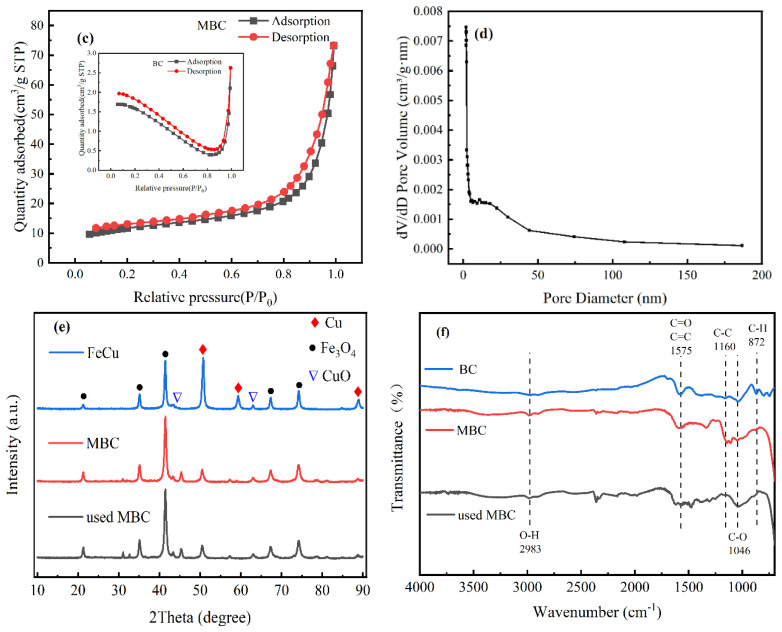
The SEM images of (**a**) BC and (**b**) MBC; (**c**) Nitrogen adsorption–desorption isotherms of MBC and BC (inset); (**d**) the corresponding pore size distribution of MBC; (**e**) XRD pattern; (**f**) FTIR spectra.

The typical SEM image of BC was revealed in Figure 2a, which observed a fold structure with a loose and porous surface. Therefore, a favorable condition can be provided for the loading of Fe and Cu. The surface of MBC was unevenly covered with the crystal of different size in Figure 2b. It indicated that the metals were successfully loaded during the preparation process.

As shown in Figure 2c, the nitrogen adsorption–desorption isotherm of MBC showed the phenomenon of adsorption lag in the middle segment, which conformed to the characteristic of IV isotherm [30]. The average pore diameter of MBC was below 10 nm (Figure 2d). These indicated that MBC was mesoporous materials [31]. It was characterized by high specific surface area and regular pore structure, which played an important role in the adsorption and catalytic reaction of pollutants. As depicted in Table 1, the SBET of BC and MBC obtained from nitrogen isotherms was 0.736 and 39.029 m^2^/g, and the average pore size was 3.805 and 9.986 nm, respectively. Compared with BC, MBC had a higher specific surface area and a more developed pore structure, which was conducive to the adsorption of CIP after modification.

#### 3.1.2. XRD Analysis

In Figure 2e, the diffraction peaks of FeCu at 21.213°, 35.053°, 41.362°, 43.378°, 67.206° and 74.124° matched perfectly with ferroferric oxide (Fe_3_O_4_, JCPDS card No. 88-0315). In addition, three characteristic peaks of 50.683°, 59.272° and 88.742° were clearly observed, which perfectly matched with copper (Cu, JCPDS card No. 85-1326). Compared with FeCu, the intensity of peaks of Cu reduced in MBC, and three new diffraction peaks appeared (2θ = 45.308°, 62.889° and 81.107°), which were consistent with copper oxide (CuO, JCPDS card No. 72-0629). These results demonstrated that the metals were successfully loaded, and MBC contained Fe_3_O_4_, CuO, a small amount of Cu. Therefore, MBC had magnetic property due to the presence of Fe_3_O_4_. In addition, the used MBC had the same diffraction pattern as MBC, indicating that the crystal structure of the catalyst remained good, which was conducive to reuse of the materials.

#### 3.1.3. FTIR Analysis

In Figure 2f, functional groups in BC and MBC were shown below: The stretching vibration band of hydroxyl groups (-OH) in carboxylic and phenolic related to MBC at 2983 cm^−1^ [32]. The absorption peak at 1575 cm^−1^ could be ascribed to the combined effect of C=O and aromatic C=C bonds. The peaks at 1160 cm^−1^ and 1046 cm^−1^ of the sample were formed by C-C and C-O bonds stretching, respectively [33,34]. The absorption peak at 872cm^−1^ represented the bending vibration of aromatic C-H bond [35]. It indicated that there were oxygen-containing functional groups in MBC. Compared with BC, the peak intensity of C=O and C=C bonds in MBC were significantly reduced. Therefore, it could infer that the oxygen-containing functional groups of BC played a role in the synthesis of MBC. Compared with MBC, it revealed that the peak strength of used MBC at 1575 cm^−1^ was significantly decreased and the peak strength at 1046 cm^−1^ was increased. It was speculated that the O=C-O groups were main reactive sites, and a part of C-O groups was generated during the catalytic reaction.

#### 3.1.4. XPS Analysis

**Figure 3:**
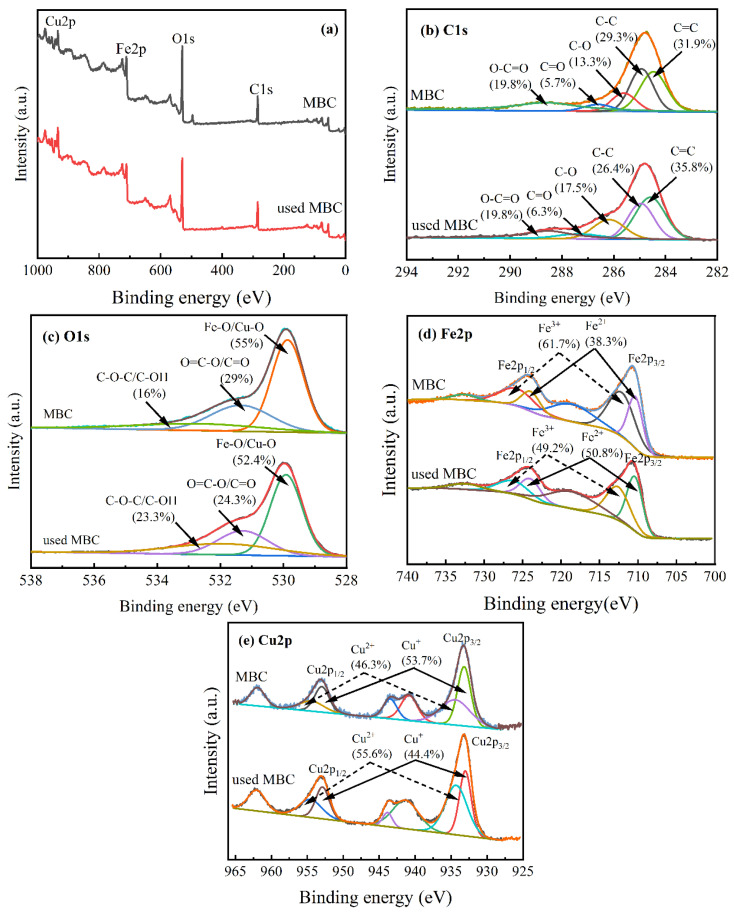
(**a**) Wide-scan XPS spectra, and high-resolution XPS spectra of MBC and used MBC: (**b**) C1s; (**c**) O1s; (**d**) Fe2p; (**e**) Cu2p.

The XPS survey spectra (Figure 3a) clearly indicated that MBC was mostly composed of C, O, Fe, and Cu. Figure 3b indicated the high-resolution XPS spectra of C1s of MBC and used MBC. C1s spectrum of catalyst could be fitted with five peaks at 284.8, 285.2, 286.6, 286 and 288.9 eV, which were associated with sp^2^C=C, sp^3^C-C, C-O, C=O and O-C=O, respectively [36]. The spectrum indicated the existence of sp^2^ C=C and sp^2^ C-C in MBC. This was in consistent with the FTIR analysis. Furthermore, it showed that graphite and amorphous carbon were present in MBC. It was beneficial to the adsorption and catalytic reactions [37,38]. In Figure 3c, the O1s spectrum of MBC and used MBC could be mainly deconvoluted into three peaks, in which at 529.9, 531.4 and 532.8 eV were assigned to Fe-O/Cu–O, O=C-O/C=O and C-O-C/C-OH, respectively [39].

The spectra of Fe2p and Cu2p were shown in Figure 3d,e, respectively, to study the Fe and Cu components in detail. Banding energies at 710.4 eV (Fe^2+^) and 712.1 eV (Fe^3+^) were attributed to Fe2p_3/2_, whereas the peaks at 724.0 eV (Fe^2+^) and 725.7 eV (Fe^3+^) were ascribed to Fe2p_1/2_ (Figure 3d). The results suggested the existence of Fe_3_O_4_. It showed that Fe^3+^ and Fe^2+^ were 61.7% and 38.3% before the reaction, respectively, and Fe^3+^ and Fe^2+^ were 49.2% and 50.8% after the reaction. Therefore, it can be seen that 12.5% of Fe^3+^ was reduced to Fe^2+^ in the reaction process. In Figure 3e, Cu 2p core level spectra of MBC could be fitted with seven peaks, in which the peaks at 933.2 eV and 952.9 eV were ascribed to the 2p_3/2_ and 2p_1/2_ binding energy of Cu^+^, respectively. Deconvolution of the Cu^2+^ peak obtained binding energies at 934.4 eV (Cu2p_3/2_) and 954.4 eV (Cu2p_1/2_). According to the change of the Cu2p peak area, 9.3% Cu^+^ was oxidized to Cu^2+^ after the reaction. These results indicated that iron and copper of MBC were involved in the electron transfer process. The existence of electron transfer can be attributed to the transformation of functional groups and the valence change of metal.

#### 3.1.5. VSM Analysis

**Figure 4:**
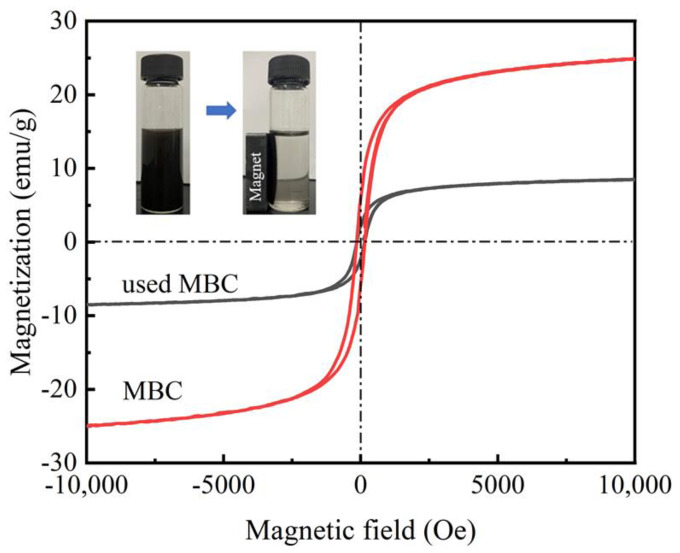
Magnetization curves of MBC and used MBC. The illustration is separation of used MBC from the solution under external magnetic field.

It was obvious that the room temperature magnetization curves of samples showed normal hysteresis loops in Figure 4. The loop shape was symmetric to the origin. The values of coercivity were basically the same as remanence. These hysteresis loops demonstrated that the saturation magnetization of MBC and used MBC were 25 and 8.5 emu/g, respectively. The reduction of used MBC was probably due to iron oxides deposition on the surface of catalysts in Fenton-like system. However, the used MBC can still be rapidly separated from the solution under the action of external magnetic field. It showed that both MBC and used MBC had good magnetic properties.

### 3.2. The Confirmation of Optimal Preparation Scheme

#### 3.2.1. Effect of Different Mass of Biomass

**Figure 5:**
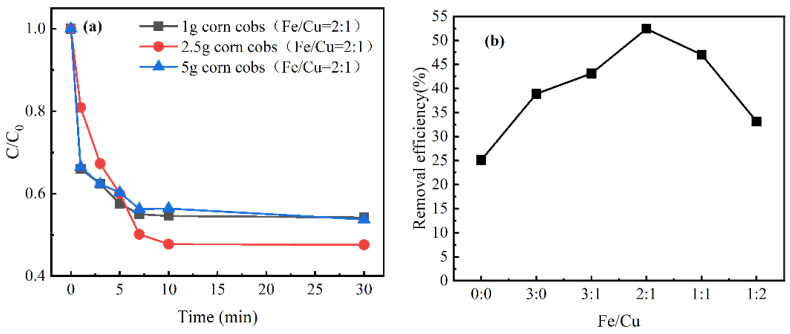
(**a**) Removal of CIP at various mass of biomass. (**b**) Removal of CIP at different Fe/Cu molar ratios. Reaction conditions: pH, 6.4; initial CIP, 10 mg/L; catalyst dosage, 0.4 g/L.

Adsorption experiments with different biomasses were conducted to explore the effect of biomass mass on adsorption efficiency. In Figure 5a, as the increase in corncob mass, the adsorption efficiency increased first and then decreased. A total of 2.5 g corncobs (52%) was better than 1 g and 5 g (46%) on the CIP removal efficiency. The above results may be because a large number of metals were difficult to separate when the mass of corncob was small. Therefore, it is inferred that metal agglomeration occupied and blocked adsorption sites, which affected the adsorption of CIP. When the mass of corncob increased, the surface area of the material increased, resulting in an increase in adsorption sites. When the mass continued to increase, the activity of catalyst was changed. The reason was that the decrease in the metal-to-biomass ratio resulted in a decrease in the number of active sites. Based on the above results, 2.5 g corncobs were selected as an optimal biomass in this study.

#### 3.2.2. Effect of Different Fe/Cu molar Ratios

The adsorption efficiency of CIP fluctuated in Figure 5b. It showed that with the increase in Cu proportion, the CIP removal efficiency noticeably increased from 25% to 52%, which may be due to the presence of copper to facilitate the electron transfer leading to the increase in adsorption [40]. However, when the Fe/Cu molar ratio exceeded 2:1, the removal efficiency of CIP decreased sharply, which may be because excessive copper block and occupy the adsorption sites except the optimal metal ratio [35]. It indicated that the presence of Cu and different Fe/Cu molar ratios had significant effects on the removal efficiency of CIP. Noticeably, the highest removal efficiency of 52% was achieved by a Fe/Cu molar ratio of 2:1. Therefore, the optimal Fe/Cu molar ratio was found to be 2:1 in the study and selected for subsequent experiments.

### 3.3. The Performance of Catalysts

#### 3.3.1. Removal of CIP at Various Systems

**Figure 6:**
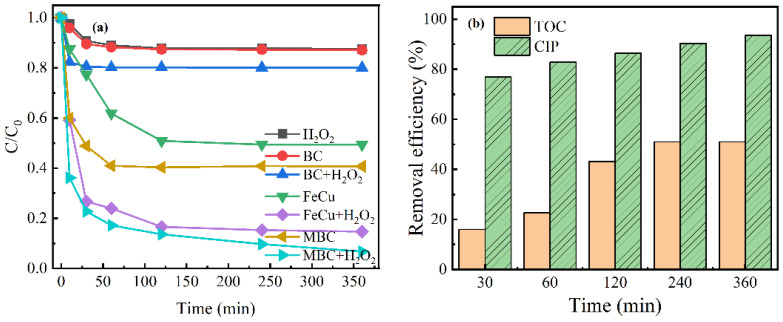
(**a**) Removal of CIP in different systems. (**b**) The removal efficiency of TOC in the MBC/H_2_O_2_. Reaction conditions: pH, 6.4; initial CIP, 10 mg/L; catalyst dosage, 0.6 g/L; H_2_O_2_, 10 mM.

The removal effects were compared under different systems to study the catalytic performance of the prepared MBC. A total of 12.4% of CIP removal was noticed in the control system with the H_2_O_2_ alone (Figure 6a). The small part of the removal was due to hydroxyl radicals produced by hydrogen peroxide self-decomposition. A total of 59.3% of CIP was adsorbed by MBC alone, whereas the adsorption efficiency of BC or FeCu alone to CIP were only 12.9% and 50.6%, respectively. MBC had a higher adsorption capacity, which may be due to its abundant pore structure. It was apparent that the decomposition efficiency of CIP was noticeably improved in the MBC/H_2_O_2_, and 93.6% of CIP was converted within 360 min, whereas 85% removal was observed for FeCu/H_2_O_2_ and only 19.9% removal for BC/H_2_O_2_. The remarkable increase in the removal effect was mainly attributed to the synergistic effect between the metal and the carbon matrix, which accelerated the decomposition for H_2_O_2_, resulting in the formation of hydroxyl radical (·OH) to degrade CIP. In addition, the conversion efficiency of TOC reached 51% within 360 min in MBC/H_2_O_2_ (Figure 6b). With the increase in CIP removal efficiency, the removal efficiency of TOC was gradually improved. Thus, it exhibits that the MBC had an excellent performance for CIP mineralization in MBC/H_2_O_2_.

#### 3.3.2. Relationship between Adsorption and Catalytic Oxidation

**Figure 7:**
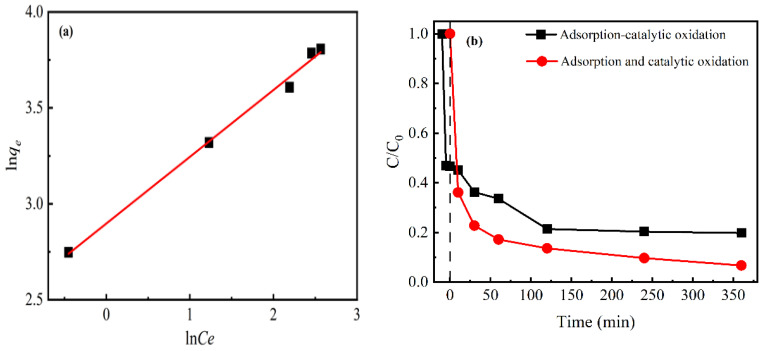
(**a**) Adsorption isotherm of CIP on the MBC. (**b**) The removal efficiency of CIP was compared between adsorption–catalytic oxidation process and adsorption and catalytic oxidation process. Reaction conditions: pH = 6.4; initial CIP, 10 mg/L; catalyst dosage, 0.6 g/L; H_2_O_2_, 10 mM.

According to the correlation coefficient (R^2^ = 0.99) of adsorption isotherm fitting, the adsorption was more consistent with the Freundlich model, indicating multilayer physical adsorption (Figure 7a). The removal of CIP was not only attributed to the adsorption of the catalyst and π–π bond interaction between the aromatic rings of CIP and carbon matrix, but also the oxidation of ·OH produced through activating H_2_O_2_. The experiments of removal CIP were performed to detect the relationship of adsorption and catalytic oxidation through changing the dosing point of hydrogen peroxide. As depicted in Figure 7b, the removal efficiency of simultaneous adsorption and the catalytic oxidation experiment (marked as adsorption and catalytic oxidation) was higher than that of catalytic oxidation after pre-adsorption (marked as adsorption–catalytic oxidation). In the adsorption and oxidation experiments, firstly, hydrogen peroxide was activated to largely generate ·OH around the catalyst. Subsequently, the rapid removal of pollutants by ·OH was due to the driving force of adsorption on MBC. Therefore, the synergistic effect between adsorption and catalytic oxidation caused a more effective removal of pollutants [41]. However, the removal efficiency of CIP was lower during the process of adsorption–catalytic oxidation. It inferred that the pollutants were adsorbed to the surface of MBC through π–π bond interaction and charge-transfer, which occupied the active sites of MBC and caused the reduction of hydroxyl radicals. Thus, the competition between adsorption and catalytic oxidation sites led to non-beneficial further removal.

### 3.4. Influencing Factor Experiment

#### 3.4.1. The Effect of Hydrogen Peroxide Concentrations

**Figure 8:**
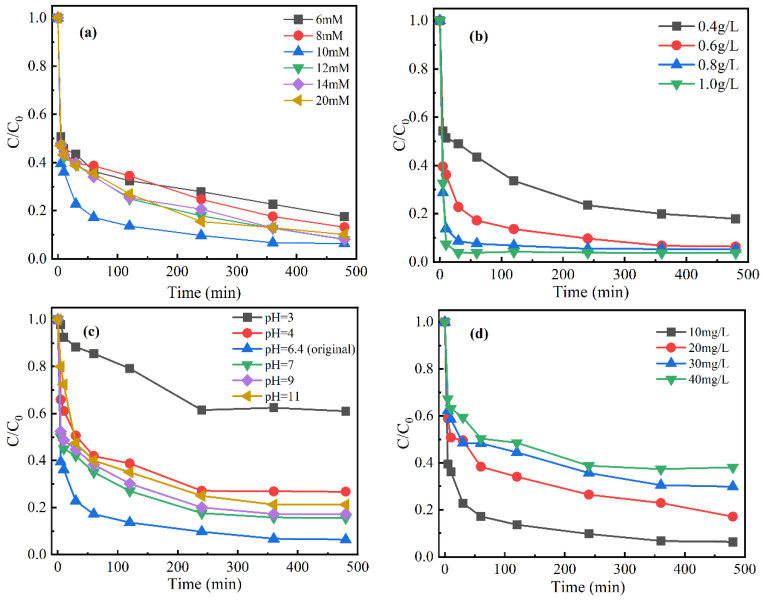
The influence of different parameters on the CIP removal in MBC/H_2_O_2_: (**a**) H_2_O_2_ concentration, (**b**) catalyst dosage, (**c**) initial pH value, and (**d**) initial CIP concentration. Except for the studied parameter, the other settings were as follows: pH, 6.4; initial CIP, 10 mg/L; catalyst dosage, 0.6 g/L; H_2_O_2_, 10 mM.

The concentration of H_2_O_2_ was 6–20 mM. According to Figure 8a, in the MBC/H_2_O_2_ condition, the removal efficiency of CIP significantly increased from 82% to 93.6% with the initial concentration of H_2_O_2_ from 6 to 10 mM after 360 min. The reason for the positive effect was that the increased H_2_O_2_ could be activated to produce more OH by MBC, which played a dominant role in the removal of CIP. However, the CIP removal efficiency decreased slightly when the H_2_O_2_ concentration continued to increase to 12 mM, 14 mM, and 20 mM. The above results were attributed to the excessive H_2_O_2_ caused the self-scavenging of OH as described by Equations (1) and (2). In addition, according to the hydrogen peroxide influencing factor test, the optimal reaction condition (10 mM) was selected for the mineralization test. It can be seen that the conversion efficiency of TOC reached 51% within 360 min in the MBC/H_2_O_2_ condition (Section 3.3.1).

H_2_O_2_ + ·OH → HO_2_· + H_2_O; (1)

HO_2_· + ·OH → H_2_O + O_2_. (2)

#### 3.4.2. The Effect of Catalyst Dosages

The MBC dosage was 0.4–1.0 g/L. As shown in Figure 8b, when the dosage was increased from 0.4 g/L to 1.0 g/L, the removal efficiency increased from 83% to 96%. The reason was that the higher catalyst dosage could provide more sites for catalytic reaction, thus producing more ·OH to be thorough on the CIP removal. It was also enhanced the release of copper and iron ions into the solution, which accelerated the reaction with H_2_O_2_, resulting in further production of ·OH. However, it could be clearly seen that the final removal efficiency changed little, and the equilibrium reaction time was significantly shortened with the dosage increase from 0.6 g/L to 1.0 g/L. The reason for the above results was the increase in adsorption sites with the increase in catalyst dosage, but ·OH may be self-scavenging due to the undesirable reaction. A total of 0.6 g/L was used as the best dosage of catalyst in subsequent experiments to reduce the amount of catalyst and save costs.

#### 3.4.3. The Effect of pH

Compared with the original pH = 6.4, the removal efficiency of CIP was reduced under other conditions (Figure 8c). With the pH values at 3–7, the removal efficiency was decreased. The main reason was that hydrogen ions cleaned up ·OH as well as the consumption of Cu^+^ caused by reacted with molecular oxygen in acidic conditions [35]. In particular, the removal efficiency significantly decreased to only 38% at the pH of 3. In addition to the above possible reasons, the adsorption of the catalyst played an important role in the removal of pollutants. The adsorption mechanism of materials was influenced by pH on electrostatic interaction [42]. The potential of zero charge (pH_pzc_) of MBC was 9.0 (Figure S1). The surface of MBC was a positive charge at pH = 3.0 (pH < pH_pzc_). The pK_a_ of CIP molecule is pK_a1_ = 6.8 and pK_a2_ = 8.7 [43]. CIP molecules existed in the form of cation at pH < 6.8. Therefore, the significant decrease in decomposition efficiency was due to the electrostatic repulsion at pH = 3.0 [44].

In addition, it can be seen that the removal efficiency of CIP in 360 min decreased slightly from 84.5% to 78.8% in the initial pH range of 7–11. It could be ascribed to the change of the decomposition pathway of H_2_O_2_ with the increase in the pH Equation (3) which tended to produce O_2_ instead of ·OH [45]. The slight reduction of the oxidation potential of ·OH may also be the reason for the decrease in removal efficiency. Compared with other catalysts, it was found that MBC had a good catalytic performance on the removal of CIP in a wider pH range (4.0–11.0) [42].

2H_2_O_2_ → 2H_2_O + O_2_; (3)

#### 3.4.4. The Effect of Initial CIP Concentrations

In Figure 8d, the CIP removal efficiency was above 70% in the initial CIP concentration range of 10–40 mg/L. The removal efficiency of CIP gradually decreased with the increase in initial pollutant concentration. The decrease in removal efficiency could be related to the occupation of active sites of MBC at high amounts of CIP [46]. In addition, the possible reason was that the ratio of ·OH to CIP decreased and the concentration of intermediate increased, leading to competition with CIP for ·OH and adsorption sites.

### 3.5. Effect of Background Components in Practical Aquatic

The organic and inorganic substances of natural water had different effects on the activation of H_2_O_2_. The influence of common anions (Cl^−^, NO_3_^−^, HCO_3_^−^, H_2_PO_4_^−^) on MBC to degrade CIP were revealed in Figure S2a–d. The removal efficiency of CIP was decreased with the concentration of anions increased, which demonstrated that anions had a negative effect in the MBC/H_2_O_2_. The reason was that these anions were oxidized by ·OH to weaker oxidation capacity radical Equations (4)–(7) [47,48]. It resulted in a slight reduce on the removal efficiency of CIP. Additionally, the same concentration of inorganic anions had different inhibition effects on the removal of CIP in the following order (Figure S2e): HCO_3_^−^ > Cl^−^> NO_3_^−^ > H_2_PO_4_^−^ > no anions. HCO_3_^−^ had the greatest influence. The reason was that the pH value of the solution increased with its concentration increased, thus reducing the removal efficiency of CIP.

Humic acid (HA) was used as a typical natural organic matter (NOM) to explore the effect of NOM on the removal of CIP in MBC/H_2_O_2_. When the concentration of HA was 2.5–10.0 mg/L, the decomposition efficiency for CIP was decreased from 93% to 69.2% (Figure S2f). It revealed that HA had a strong influence on the removal of CIP. The reason was that the oxygen-containing functional group of HA not only competed with H_2_O_2_ for active sites, but also with the pollutant for ·OH [49]. In addition, it may be due to the complex formation between CIP molecules and humic acid, which prevented their adsorption onto MBC nanomaterial [50].

Compared with ultrapure water, the removal experiments of CIP in actual water (tap water and lake water) were carried out to investigate the performance of MBC. The characteristics of actual water were shown in Table S1 [51]. Totals of 75.8% and 72.4% of CIP were removed in tap water and lake water (Figure S2g). The decrease in removal efficiency may be due to their high DOC concentrations and turbidity.

**·**OH + Cl^−^→ HOCl**·**^−^         k = 4.3 × 10^9^ M^−1^s^−1^ (4)

**·**OH + NO_3_^−^→ OH^−^+ NO_3_**·**     k < 5.0 ×10^5^ M^−1^s^−1^ (5)

**·**OH + H_2_PO_4_^−^ → OH^−^ + H_2_PO_4_**·**     k = 2.0 × 10^4^ M^−1^s^−1^ (6)

**·**OH + HCO_3_^−^ → H_2_O + HCO_3_**·**    k = 1.6 × 10^6^ M^−1^s^−1^ (7)

### 3.6. Reusability and Stability of MBC

**Figure 9:**
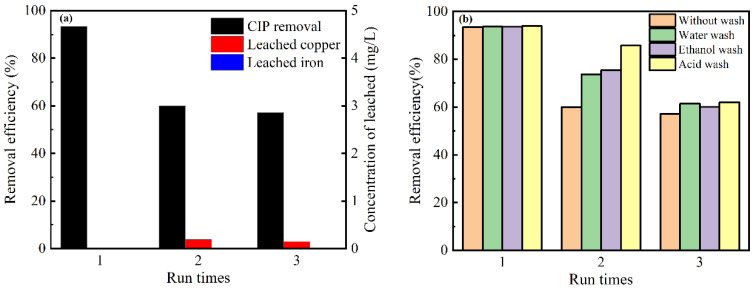
(**a**) Removal efficiency of CIP and the corresponding coppers and irons leaching concentration without washing in three cycles. (**b**) Three consecutive experiments under four conditions.

An external magnetic field separated the catalyst from the solution and then reused directly without washing. Three cycle experiments were performed under the same conditions to explore the stability of the catalyst. According to Figure 9a, it can be seen that the CIP removal degree gradually decreased with the increase in reactions. In the third cycle, the performance of the used MBC was 61% of the MBC, indicating that the catalyst still maintained a certain catalytic activity. It could be found that almost no iron was leached after three cycle reactions (Figure 9a), which is much lower than that of some iron oxide catalysts and zero-valent iron [52,53]. The concentration of leached copper was in the range of 0~0.1905 mg/L. This result indicated that the mesoporous carbon matrix of MBC can play a role in a barrier to alleviate the metal leached.

The removal CIP of recycling experiments were compared under four conditions to further evaluate the reusability of MBC. Figure 9b revealed that, compared with ethanol and water, the removal of CIP was increased by using an acid wash catalyst. The above results may be caused by oxide generation, which will hinder the occurrence of Fenton-like reactions. The catalyst after an acid wash could dissolve the oxides on the surface.

### 3.7. The Removal Mechanisms

#### 3.7.1. The Contribution of Adsorption and Degradation

The desorption experiments were performed to study the contribution of adsorption and catalytic oxidation on the removal of CIP. After 360 min of the reaction in the MBC/H_2_O_2_ condition, the catalyst was separated by an external magnetic field, and then 0.1 M NaOH solution was used as the desorption experiment for 24 h [30]. In the end, the absorbance was measured to obtain the adsorption efficiency. The contribution of adsorption and catalytic oxidation were 31.7% and 61.3% on the removal efficiency of CIP, respectively. According to calculation, the catalytic oxidation efficiency of CIP accounted for 66%, whereas the adsorption efficiency was 34% in the removal.

#### 3.7.2. Determination of Free Radicals in the Degradation Process

**Figure 10:**
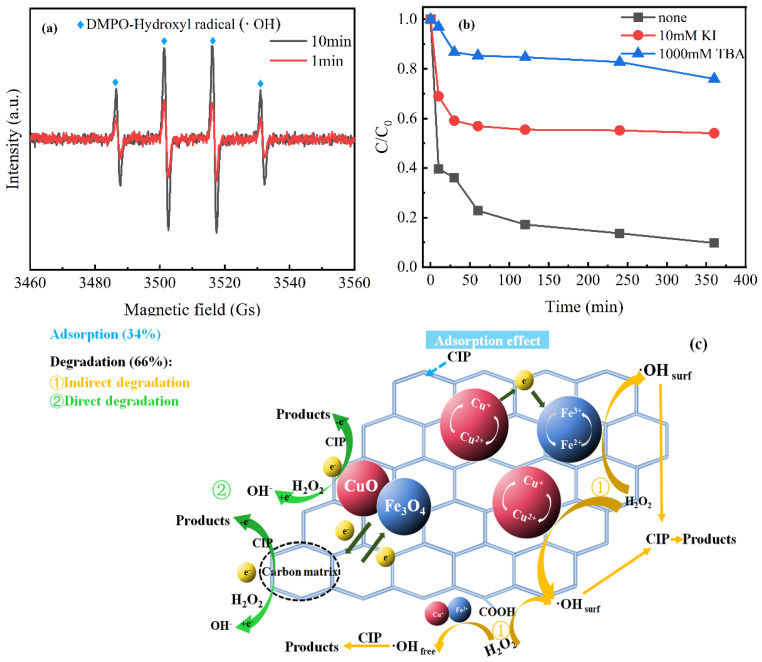
(**a**) Comparison of EPR spectra at 1min and 10min in the MBC/H_2_O_2_. (**b**) Effect of TBA or KI scavengers on the removal of CIP in the MBC/H_2_O_2_. (**c**) Mechanisms of CIP removal in the MBC/H_2_O_2_.

Due to hydroxyl radicals of DMPO capture, the free radicals generated were testified by coupling EPR with DMPO. The specific signal of DMPO-·OH of MBC was observed (Figure 10a). Compared with 1 min, DMPO-·OH specific signal of MBC was clearly visible after 10min reaction. Additionally, a representative signal of 1:2:2:1 was monitored, which represented the DMPO-·OH adduct [54]. Based on the above results, MBC activated H_2_O_2_ to produce a large amount of ·OH after 10 min, and ·OH participated in the reaction.

It was generally accepted that hydrogen peroxide was activated by the catalyst to produce the surface-bound hydroxyl radicals (on catalyst surface) (·OH_surf_) to degrade the CIP. In the solution, the leached copper and iron ions were the viable specie that could also activate H_2_O_2_ to generate free ·OH (·OH_free_). Therefore, to further distinguish whether reaction of the removal CIP was dominated by ·OH_surf_ or ·OH_free_, free radical quenching experiments were performed by adding excess TBA (1000 mM) and KI (10 mM), respectively. Excess TBA could react with both ·OH_surf_ and ·OH_free_ in the MBC/H_2_O_2_, whereas KI could only effectively scavenge the ·OH_surf_ that was generated on the surface-active sites of MBC catalyst. Figure 10b displayed that it had markedly inhibitory effects on the decomposition of CIP with addition of both TBA (31%) and KI (45%) compared with that without quenchers (93%). It showed that ·OH played a major role in the degradation of CIP and the rapid degradation of CIP was more relevant to ·OH_surf_ than ·OH_free_.

#### 3.7.3. The Possible Removal Mechanism

The possible mechanism of removal CIP in the MBC/H_2_O_2_ may be as follows (Figure 10c): When there was the existence of H_2_O_2_, copper and iron ions on the surface of MBC could catalyze H_2_O_2_ to generate ·OH_surf_ through the intermolecular electron transfer process (Equations (8)–(11)). Copper had a similar reaction to iron, which can also activate H_2_O_2_ to generate ·OH_surf_ at a higher rate constant (k = 1.0 × 10^4^ M^−1^s^−1^) than iron (k = 63 − 76 M^−1^s^−1^) [55,56]. At the same time, there was electron transfer from ≡Cu^+^ to ≡Fe^3+^, which might regenerate ≡Fe^2+^ and ≡Cu^2+^ via the Haber–Weiss reaction (Equation (12)) [57]. It was conducive to the redox cycles of Fe^3+^/Fe^2+^ and Cu^2+^/Cu^+^, respectively, and the continuous generation of ·OH_surf_ by activated H_2_O_2_. Moreover, the oxygen-containing functional groups of MBC could react with H_2_O_2_ to form ·OH_surf_ by means of electron transfer (Equation (13)) [58]. Meanwhile, a small amount of leached ions could activate H_2_O_2_ to produce ·OH_free_ through chain reaction (Equations (15)–(18)). Finally, CIP was degraded by ·OH_surf_ generated on the surface of MBC (Equations (14)) and a small part of ·OH_free_ in the solution (Equation (19)).

≡Fe^2+^ + H_2_O_2_ → ≡Fe^3+^ + ·OH_surf_ + OH^−^; (8)

≡Fe^3+^ + H_2_O_2_ → ≡Fe^2+^ + HO_2_·_surf_ + H^+^; (9)

≡Cu^2+^ + H_2_O_2_ → ≡Cu+ + HO_2_·_surf_ + H^+^; (10)

≡Cu^+^ + H_2_O_2_ → ≡Cu^2+^ + ·OH _surf_ + OH^−^; (11)

≡Fe^3+^ + ≡Cu^+^ → ≡Fe^2+^ + ≡Cu^2+^; (12)

BC-O-C=O + H_2_O_2_ → BC^+^ + OH^−^ + ·OH _surf_ (13)

CIP+ ·OH _surf_ → intermediates → CO_2_ + H_2_O. (14)

Fe^2+^ + H_2_O_2_ → Fe^3+^ + ·OH_free_ + OH^−^; (15)

Fe^3+^ + H_2_O_2_ → Fe^2+^+ HO_2_·_free_ + H^+^; (16)

Cu^2+^ + H_2_O_2_ → Cu^+^+ HO_2_·_free_ + H^+^; (17)

Cu^+^ + H_2_O_2_ → Cu^2+^+ ·OH _free_ + OH^−^; (18)

CIP + ·OH _free_ →intermediates → CO_2_ + H_2_O. (19)

CIP was not only degraded indirectly through the oxidation of ·OH, but also directly by electron transfer processes of the oxygen-containing functional groups and metals in MBC. As shown in the C1s XPS spectra (Figure 3b), the percentage of the main reactive sites O=C-O decreased from 19.8% to 14%, whereas the C=O increased from 5.7% to 6.3%. It was inferred that C=O were produced by O=C-O due to electron transfer processes [36]. The carbon base acted as a medium for electron transfer from CIP (electron donor) to H_2_O_2_ (electron acceptor) [59]. Meanwhile, in the O1s XPS spectra (Figure 3c), the decrease in Fe-O/Cu-O from 55% to 52.4% showed that the electron transfer of iron and copper also contributed to the degradation of CIP [30].

### 3.8. The Intermediate Products and Possible Degradation Pathway

#### 3.8.1. The Intermediate Products

**Figure 11:**
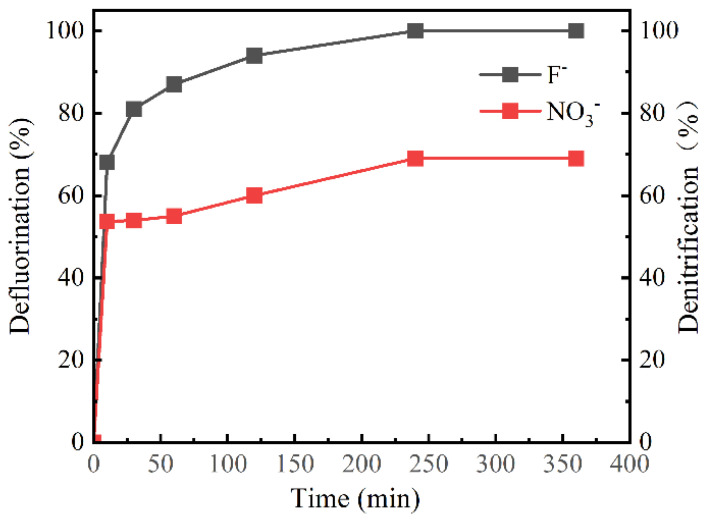
Defluorination and denitrification efficiencies in the MBC/H_2_O_2_ condition. Reaction conditions: pH = 6.4; initial CIP, 10 mg/L; catalyst dosage, 0.6 g/L; H_2_O_2_, 10mM.

CIP and the intermediates in the MBC/H_2_O_2_ were examined by HPLC-MS and speculated based on previous research (Figure S3) [8,28,60]. Ten intermediate products were detected and displayed in Table S2. At the same time, F^−^ and NO^3−^ were detected by IC in 20 min. In Figure 11, the fluorine atoms were almost entirely converted to F^−^ and the defluoridation efficiency reached 100%. N atoms were not completely converted to NO^3Ȓ^. It can be inferred that N-containing intermediates and other inorganic ions containing N atoms were formed during the whole degradation process.

Moreover, Gaussian 09 was used to optimize the spatial geometry of CIP and predict the attack sites of hydroxyl radical on CIP. As shown in Figure S4, the optimized conformation of CIP molecule was obtained by DFT at the B3LYP/6-31+ G* level. The bond length and atomic natural charge in CIP structure were listed in Table S3. With the bond length increased, the bond energy was decreased. In the process of oxidative degradation of antibiotics, bonds with low bond energy are easy to break [61]. O(26), N(13), N(42), and N(41) had higher negative charges than others in CIP, thus they were easily attacked by ·OH. In addition, the longer bonds were more easily broken, such as C(30)-C(28), C(15)-N(13), F(14)-C(6), N(41)-C(29), N(41)-C(28), C(11)-C(10) and C(8)-C(4) bonds, resulting in intermediates. All above the analyses were consistent with the results of HPLC-MS.

#### 3.8.2. Possible Degradation Pathway

**Figure 12:**
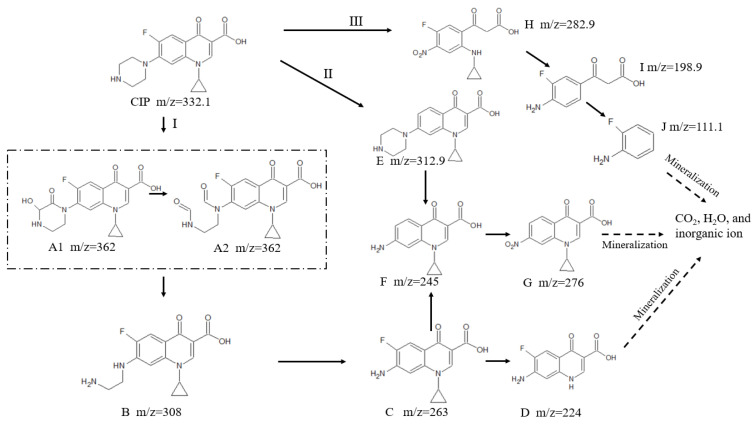
Degradation pathways of the CIP degradation products.

Based on HPLC-MS, IC and the Gaussian calculations, there were three possible degradation pathways of CIP as follows: pathway I, piperazine ring cleaved; pathway II, defluorination; pathway III, pyridine ring cleaved (Figure 12). The ring rupture caused by piperazine epoxidation was an important pathway to remove CIP, with relatively high abundances of the products A and B [62]. In pathway I, ·OH can oxidize the secondary amine of the piperazine ring to obtain the compound A1 (m/z = 362). The product A2 was formed by the alcohol group oxidized to the carbonyl group (-C=O) and the piperazine ring cleaved to the aldehyde group (-CHO). Subsequently, the product A can be decarbonylation, resulting in product B (m/z = 308). CIP could also be directly converted into product B under the action of ·OH. The reason was that N atoms of piperazine ring were higher negative charges according to the Gaussian calculations, thus it was easily attacked by ·OH. The product C containing aniline (m/z = 263) was generated by losing the N atom, oxidation to ketone, and then a decarbonylation reaction. At this point, the piperazine ring of CIP was completely destroyed. Product C moved the cyclopropyl into product D (m/z = 224), and product F (m/z = 245) can also be generated by defluorination. In pathway II, since the F(14)-C(6) of the benzene ring was longer bond length, it was more vulnerable to be attacked by ·OH to form the product E (m/z = 312.9). The product E was further oxidized to generate product F (m/z = 245), similar to the conversion of CIP to product C and product G (m/z = 276). Pathway III mainly consisted of the cleavage of piperazine and pyridine rings, resulting in product J (m/z = 111.1). Additionally, J, G, and D then had a chain of oxidation reactions and eventually mineralized into small molecules and inorganic ions [63].

## 4. Conclusions

In summary, a novel corncob biochar-based magnetic iron–copper bimetallic nanomaterial catalyst (MBC) was successfully synthesized by the co-precipitation method, and the removal of ciprofloxacin was realized. The removal efficiency of CIP was 93.6% within 360 min, using a H_2_O_2_ dose of 10 mM and an MBC concentration of 0.6 g/L at pH 6.8 in MBC/H_2_O_2_. The high removal efficiency of CIP was not only due to the adsorption of MBC (34%), but also due to direct and indirect degradation (66%). MBC had a good catalytic performance in a wider pH range (4.0–11.0). The inorganic anions of natural water had different inhibition effects on the removal of CIP in the following order: HCO_3_^−^ > Cl^−^> NO_3_^−^ > H_2_PO_4_^−^ > no anions. Moreover, HA had a strong influence on the removal of CIP. EPR and quenching experiments validated that ·OH was the dominant reactive species on the degradation of CIP. The electron transfer and redox cycles between metal ions and the synergistic action between catalytic degradation and adsorption contributed to the removal of CIP. Furthermore, through the analysis of HPLC-MS, IC and the Gaussian calculations, several intermediate products had been found. Additionally, it was inferred that there were three possible pathways in CIP degradation process in this work: pathway I, piperazine ring cleaved; pathway II, defluorination; pathway III, pyridine ring cleaved. As it can be seen, there are still some metal ions leaching with the reaction, resulting in a slight loss of stability of MBC. Therefore, we will continue to study root cause of metal leaching, so as to further improve the stability of the catalyst. These findings can provide great scalability for the preparation of using other waste biomass as catalysts to improve the removal of organic pollutants from aqueous solutions.

## Figures and Tables

**Table 1:** Structural parameters of BC and MBC.

Samples	S_BET_ (m^2^/g)	Average Pore (nm)
BC	0.736	3.805
MBC	39.029	9.986

## Data Availability

The data is not available due to [certain reason, privacy, further study, and so on].

## Supplementary Materials

The following are available online at https://www.mdpi.com/article/10.3390/nano12040579/s1. Text S1: List of chemical reagents; Text S2: Characterization; Text S3: Analytic methods. Figure S1: Zeta potential of MBC. Figure S2: Effects of inorganic anions: (**a**) Cl^−^, (**b**) NO_3_^−^, (**c**) HCO_3_^−^, (**d**) H_2_PO_4_^−^, (**e**) Comparison of four inorganic ions; (**f**) Effect of HA; (**g**) CIP removal in actual water (Reaction conditions: pH, 6.4; initial CIP, 10 mg/L; MBC dosage, 0.6 g/L; H_2_O_2_, 10 mM; temperature, 25 °C). Figure S3: Mass spectrogram in the MBC/H_2_O_2_ condition. Figure S4: The optimized conformation of CIP with atomic labeling. Table S1: Characteristics of the water samples. Table S2: Degraded products of CIP by catalytic hydrogen peroxide decomposition. Table S3: The atomic natural charge and bond length of CIP molecule.

## Abbreviations

MBC	Corncob biochar-based magnetic iron–copper bimetallic nanomaterial
CIP	Ciprofloxacin
TOC	Total organic carbon
AOPs	Advanced oxidation processes
SEM	Scanning electron microscopy
XRD	X-ray diffraction
FTIR	Fourier-transform infrared spectroscopy
XPS	X-ray electron spectroscopy
VSM	Vibration sample magnetometer
EPR	Electron paramagnetic resonance
DFT	Density functional theory
TBA	Tert-butanol alcohol

## References

[1] Yu J., Tang L., Pang Y., Zeng G., Wang J., Deng Y., Liu Y., Feng H., Chen S., Ren X. (2019). Magnetic nitrogen-doped sludge-derived biochar catalysts for persulfate activation: Internal electron transfer mechanism. Chem. Eng. J..

[2] Wang J., Tang L., Zeng G., Deng Y., Liu Y., Wang L., Zhou Y., Guo Z., Wang J., Zhang C. (2017). Atomic scale g-C_3_N_4_/Bi_2_WO_6_ 2D/2D heterojunction with enhanced photocatalytic degradation of ibuprofen under visible light irradiation. Appl. Catal. B Environ..

[3] Deng Y., Tang L., Zeng G., Zhu Z., Yan M., Zhou Y., Wang J., Liu Y., Wang J. (2017). Insight into highly efficient simultaneous photocatalytic removal of Cr(VI) and 2,4-diclorophenol under visible light irradiation by phosphorus doped porous ultrathin g-C_3_N_4_ nanosheets from aqueous media: Performance and reaction mechanism. Appl. Catal. B Environ..

[4] Zhang X., Li Y., Wu M., Pang Y., Hao Z., Hu M., Qiu R., Chen Z. (2021). Enhanced adsorption of tetracycline by an iron and manganese oxides loaded biochar: Kinetics, mechanism and column adsorption. Bioresour. Technol..

[5] Shah N.S., Ali Khan J., Sayed M., Ul Haq Khan Z., Sajid Ali H., Murtaza B., Khan H.M., Imran M., Muhammad N. (2019). Hydroxyl and sulfate radical mediated degradation of ciprofloxacin using nano zerovalent manganese catalyzed S_2_O_8_^2−^. Chem. Eng. J..

[6] Iqbal J., Shah N.S., Sayed M., Muhammad N., Rehman S., Khan J.A., Haq Khan Z.U., Howari F.M., Nazzal Y., Xavier C. (2020). Deep eutectic solvent-mediated synthesis of ceria nanoparticles with the enhanced yield for photocatalytic degradation of flumequine under UV-C. J. Water Process Eng..

[7] Deng W., Li N., Zheng H., Lin H. (2016). Occurrence and risk assessment of antibiotics in river water in Hong Kong. Ecotox. Environ. Safe..

[8] Li J., Pan L., Yu G., Xie S., Li C., Lai D., Li Z., You F., Wang Y. (2019). The synthesis of heterogeneous Fenton-like catalyst using sewage sludge biochar and its application for ciprofloxacin degradation. Sci. Total Environ..

[9] Patel M., Kumar R., Kishor K., Mlsna T., Pittman C.J., Mohan D. (2019). Pharmaceuticals of Emerging Concern in Aquatic Systems: Chemistry, Occurrence, Effects, and Removal Methods. Chem. Rev..

[10] Rahim Pouran S., Abdul Raman A.A., Wan Daud W.M.A. (2014). Review on the application of modified iron oxides as heterogeneous catalysts in Fenton reactions. J. Clean. Prod..

[11] Yin D., Xu Z., Shi J., Shen L., He Z. (2018). Adsorption characteristics of ciprofloxacin on the schorl: Kinetics, thermodynamics, effect of metal ion and mechanisms. J. Water Reuse Desal..

[12] Alnajrani M.N., Alsager O.A. (2020). Removal of Antibiotics from Water by Polymer of Intrinsic Microporosity: Isotherms, Kinetics, Thermodynamics, and Adsorption Mechanism. Sci. Rep..

[13] Jiang S., Zhu J., Wang Z., Ge M., Zhu H., Jiang R., Zong E., Guan Y. (2018). Efficiency and Mechanism of Ciprofloxacin Hydrochloride Degradation in Wastewater by Fe_3_O_4_/Na_2_S_2_O_8_. Ozone Sci. Eng..

[14] Chen J., Liu Y., Zhang J., Yang Y., Hu L., Yang Y., Zhao J., Chen F., Ying G. (2017). Removal of antibiotics from piggery wastewater by biological aerated filter system: Treatment efficiency and biodegradation kinetics. Bioresour. Technol..

[15] Chen H., Gao B., Li H. (2014). Functionalization, pH, and ionic strength influenced sorption of sulfamethoxazole on graphene. J. Environ. Chem. Eng..

[16] Aseman-Bashiz E., Rezaee A., Moussavi G. (2021). Ciprofloxacin removal from aqueous solutions using modified electrochemical Fenton processes with iron green catalysts. J. Mol. Liq..

[17] Yang H., Zhou M., Yang W., Ren G., Ma L. (2018). Rolling-made gas diffusion electrode with carbon nanotube for electro-Fenton degradation of acetylsalicylic acid. Chemosphere.

[18] Hassani A., Karaca M., Karaca S., Khataee A., Açışlı Ö., Yılmaz B. (2018). Preparation of magnetite nanoparticles by high-energy planetary ball mill and its application for ciprofloxacin degradation through heterogeneous Fenton process. J. Environ. Manage..

[19] Rani V., Das R.K., Golder A.K. (2017). Fabrication of reduced graphene oxide-graphite paste electrode for H_2_O_2_ formation and its implication for ciprofloxacin degradation. Surf. Interfaces.

[20] Lima V.B., Goulart L.A., Rocha R.S., Steter J.R., Lanza M.R.V. (2020). Degradation of antibiotic ciprofloxacin by different AOP systems using electrochemically generated hydrogen peroxide. Chemosphere.

[21] Mdletshe L.S., Makgwane P.R., Ray S.S. (2019). Fabrication of Bimetal CuFe_2_O_4_ Oxide Redox-Active Nanocatalyst for Oxidation of Pinene to Renewable Aroma Oxygenates. Nanomaterials.

[22] Xu J., Zhang X., Sun C., Wan J., He H., Wang F., Dai Y., Yang S., Lin Y., Zhan X. (2019). Insights into removal of tetracycline by persulfate activation with peanut shell biochar coupled with amorphous Cu-doped FeOOH composite in aqueous solution. Environ. Sci. Pollut. R..

[23] Tang J., Wang J. (2018). Fenton-like degradation of sulfamethoxazole using Fe-based magnetic nanoparticles embedded into mesoporous carbon hybrid as an efficient catalyst. Chem. Eng. J..

[24] Zhang R., Li Y., Wang Z., Tong Y., Sun P. (2020). Biochar-activated peroxydisulfate as an effective process to eliminate pharmaceutical and metabolite in hydrolyzed urine. Water Res..

[25] You Y., Zhao Z., Song Y., Li J., Li J., Cheng X. (2021). Synthesis of magnetized nitrogen-doped biochar and its high efficiency for elimination of ciprofloxacin hydrochloride by activation of peroxymonosulfate. Sep. Purif. Technol..

[26] Liu C., Li B., Du H., Lv D., Zhang Y., Yu G., Mu X., Peng H. (2016). Properties of nanocellulose isolated from corncob residue using sulfuric acid, formic acid, oxidative and mechanical methods. Carbohyd. Polym..

[27] Feng Y., Zhang L., Yang Z., Fan Y., Shih K., Li H., Liu Y., Wu D. (2020). Nonradical degradation of microorganic pollutants by magnetic N-doped graphitic carbon: A complement to the unactivated peroxymonosulfate. Chem. Eng. J..

[28] Gao J., Han D., Xu Y., Liu Y., Shang J. (2020). Persulfate activation by sulfide-modified nanoscale iron supported by biochar (S-nZVI/BC) for degradation of ciprofloxacin. Sep. Purif. Technol..

[29] Xu L., Wang J. (2012). Magnetic Nanoscaled Fe_3_O_4_/CeO_2_ Composite as an Efficient Fenton-Like Heterogeneous Catalyst for Degradation of 4-Chlorophenol. Environ. Sci. Technol..

[30] Liu J., Luo K., Li X., Yang Q., Wang D., Wu Y., Chen Z., Huang X., Pi Z., Du W. (2020). The biochar-supported iron-copper bimetallic composite activating oxygen system for simultaneous adsorption and degradation of tetracycline. Chem. Eng. J..

[31] Tian Y., Zhong S., Huang A., Pan Y., Wang X. (2015). Large pore size FeNi carbon microspheres: Synthesis and tretinoin adsorption behavior. Mater. Lett..

[32] Wu Y., Li X., Yang Q., Wang D., Yao F., Cao J., Chen Z., Huang X., Yang Y., Li X. (2020). Mxene-modulated dual-heterojunction generation on a metal-organic framework (MOF) via surface constitution reconstruction for enhanced photocatalytic activity. Chem. Eng. J..

[33] Su H., Fang Z., Tsang P.E., Fang J., Zhao D. (2016). Stabilisation of nanoscale zero-valent iron with biochar for enhanced transport and in-situ remediation of hexavalent chromium in soil. Environ. Pollut..

[34] Wang K., Gao W., Jiang P., Lan K., Yang M., Huang X., Ma L., Niu F., Li R. (2019). Bi-functional catalyst of porous N-doped carbon with bimetallic FeCu for solvent-free resultant imines and hydrogenation of nitroarenes. Mol. Catal..

[35] Tang J., Wang J. (2020). Iron-copper bimetallic metal-organic frameworks for efficient Fenton-like degradation of sulfamethoxazole under mild conditions. Chemosphere.

[36] Chen F., Yang Q., Wang S., Yao F., Sun J., Wang Y., Zhang C., Li X., Niu C., Wang D. (2017). Graphene oxide and carbon nitride nanosheets co-modified silver chromate nanoparticles with enhanced visible-light photoactivity and anti-photocorrosion properties towards multiple refractory pollutants degradation. Appl. Catal. B Environ..

[37] Duan X., Ao Z., Zhang H., Saunders M., Sun H., Shao Z., Wang S. (2018). Nanodiamonds in sp^2^/sp^3^ configuration for radical to nonradical oxidation: Core-shell layer dependence. Appl. Catal. B Environ..

[38] Luo K., Yang Q., Pang Y., Wang D., Li X., Lei M., Huang Q. (2019). Unveiling the mechanism of biochar-activated hydrogen peroxide on the degradation of ciprofloxacin. Chem. Eng. J..

[39] Pi Z., Li X., Wang D., Xu Q., Tao Z., Huang X., Yao F., Wu Y., He L., Yang Q. (2019). Persulfate activation by oxidation biochar supported magnetite particles for tetracycline removal: Performance and degradation pathway. J. Clean. Prod..

[40] Ma J., Zhou B., Zhang H., Zhang W., Wang Z. (2019). Activated municipal wasted sludge biochar supported by nanoscale Fe/Cu composites for tetracycline removal from water. Chem. Eng. Res. Des..

[41] Zhang B., Wu T., Sun D., Chen W., Li G., Li Y. (2019). NH_2_-MCM-41 supported on nitrogen-doped graphene as bifunctional composites for removing phenol compounds: Synergistic effect between catalytic degradation and adsorption. Carbon.

[42] Wan Z., Wang J. (2017). Degradation of sulfamethazine using Fe_3_O_4_-Mn_3_O_4_/reduced graphene oxide hybrid as Fenton-like catalyst. J. Hazard. Mater..

[43] Olivera M.E., Manzo R.H., Junginger H.E., Midha K.K., Shan V.P., Stavchansky S., Dressman J.B., Barends D.M. (2010). Biowaiver Monographs for Immediate Release Solid Oral Dosage Forms: Ciprofloxacin Hydrochloride. J. Pharm. Sci..

[44] Inbaraj B.S., Chen B., Liao C., Chen B. (2020). Green synthesis, characterization and evaluation of catalytic and antibacterial activities of chitosan, glycol chitosan and poly(γ-glutamic acid) capped gold nanoparticles. Int. J. Biol. Macromol..

[45] Bautista P., Mohedano A.F., Casas J.A., Zazo J.A., Rodriguez J.J. (2011). Highly stable Fe/γ-Al_2_O_3_ catalyst for catalytic wet peroxide oxidation. J. Chem. Technol. Biotechnol..

[46] Aseman-Bashiz E., Rezaee A., Moussavi G. (2021). Effective removal of hexavalent chromium using microbial cellulose/polyaniline cathode and nanosized FeS_2_ in the form of an integrated electrochemical system. J. Water Process Eng..

[47] Cai W., Peng T., Yang B., Xu C., Liu Y., Zhao J., Gu F., Ying G. (2020). Kinetics and mechanism of reactive radical mediated fluconazole degradation by the UV/chlorine process: Experimental and theoretical studies. Chem. Eng. J..

[48] Yang J.E., Yuan B., Cui H., Wang S., Fu M. (2017). Modulating oxone-MnOx/silica catalytic systems towards ibuprofen degradation: Insights into system effects, reaction kinetics and mechanisms. Appl. Catal. B Environ..

[49] Liu J., Dong C., Deng Y., Ji J., Bao S., Chen C., Shen B., Zhang J., Xing M. (2018). Molybdenum sulfide Co-catalytic Fenton reaction for rapid and efficient inactivation of Escherichia coli. Water Res..

[50] Inbaraj B.S., Sridhar K., Chen B. (2021). Removal of polycyclic aromatic hydrocarbons from water by magnetic activated carbon nanocomposite from green tea waste. J. Hazard. Mater..

[51] Zhang T., Yang Y., Li X., Yu H., Wang N., Li H., Du P., Jiang Y., Fan X., Zhou Z. (2020). Degradation of sulfamethazine by persulfate activated with nanosized zero-valent copper in combination with ultrasonic irradiation. Sep. Purif. Technol..

[52] Segura Y., Martínez F., Melero J.A., Fierro J.L.G. (2015). Zero valent iron (ZVI) mediated Fenton degradation of industrial wastewater: Treatment performance and characterization of final composites. Chem. Eng. J..

[53] Xu L., Wang J. (2012). Fenton-like degradation of 2,4-dichlorophenol using Fe_3_O_4_ magnetic nanoparticles. Appl. Catal. B Environ..

[54] Liu S., Lai C., Li B., Zhang C., Zhang M., Huang D., Qin L., Yi H., Liu X., Huang F. (2020). Role of radical and non-radical pathway in activating persulfate for degradation of p-nitrophenol by sulfur-doped ordered mesoporous carbon. Chem. Eng. J..

[55] Bokare A.D., Choi W. (2014). Review of iron-free Fenton-like systems for activating H_2_O_2_ in advanced oxidation processes. J. Hazard. Mater..

[56] Pham A.N., Xing G., Miller C.J., Waite T.D. (2013). Fenton-like copper redox chemistry revisited: Hydrogen peroxide and superoxide mediation of copper-catalyzed oxidant production. J. Catal..

[57] Tian X., Jin H., Nie Y., Zhou Z., Yang C., Li Y., Wang Y. (2017). Heterogeneous Fenton-like degradation of ofloxacin over a wide pH range of 3.6–10.0 over modified mesoporous iron oxide. Chem. Eng. J..

[58] Fang G., Zhu C., Dionysiou D.D., Gao J., Zhou D. (2015). Mechanism of hydroxyl radical generation from biochar suspensions: Implications to diethyl phthalate degradation. Bioresour. Technol..

[59] Ding Y., Wang X., Fu L., Peng X., Pan C., Mao Q., Wang C., Yan J. (2021). Nonradicals induced degradation of organic pollutants by peroxydisulfate (PDS) and peroxymonosulfate (PMS): Recent advances and perspective. Sci. Total Environ..

[60] Wang B., Liu G., Ye B., Ye Y., Zhu W., Yin S., Xia J., Li H. (2019). Novel CNT/PbBiO_2_Br hybrid materials with enhanced broad spectrum photocatalytic activity toward ciprofloxacin (CIP) degradation. J. Photochem. Photobiol. A Chem..

[61] Li H., Li T., He S., Zhou J., Wang T., Zhu L. (2020). Efficient degradation of antibiotics by non-thermal discharge plasma: Highlight the impacts of molecular structures and degradation pathways. Chem. Eng. J..

[62] Li S., Huang T., Du P., Liu W., Hu J. (2020). Photocatalytic transformation fate and toxicity of ciprofloxacin related to dissociation species: Experimental and theoretical evidences. Water Res..

[63] Luo J., Bo S., Qin Y., An Q., Xiao Z., Zhai S. (2020). Transforming goat manure into surface-loaded cobalt/biochar as PMS activator for highly efficient ciprofloxacin degradation. Chem. Eng. J..

