# Supplementary material for: Adsorption and Fenton-like Degradation of Ciprofloxacin Using Corncob Biochar-Based Magnetic Iron–Copper Bimetallic Nanomaterial in Aqueous Solutions
Source: Nanomaterials (Basel). 2022 Feb 9;12(4):579. doi: 10.3390/nano12040579 (PMC8880508; doi:10.3390/nano12040579)
Supplement: Supplementary file 1 [file nanomaterials-12-00579-s001.zip › nanomaterials-1567218-supplementary.pdf]

# Supplementary materials for

## Adsorption and Fenton-Like Degradation of Ciprofloxacin Using Corncob Biochar-Based Magnetic Iron–Copper Bimetallic Nanomaterial in Aqueous Solutions


**Hongrun Liu, Yuankun Liu*, Xing Li, Xiaoying Zheng, Xiaoying Feng, Aixin Yu**

Municipal Engineering Department, College of Civil Engineering and Architecture,

Beijing University of Technology, Beijing 100124, P.R China

E-mails for all authors:

**Hongrun Liu,** liuhongrun7768@163.com

**Yuankun Liu,** liuyuankun@bjut.edu.cn

**Xing Li,** lixing@bjut.edu.cn

**Xiaoying Zheng,** zhengxiaoying@bjut.edu.cn

**Xiaoying Feng,** 18801365714@emails.bjut.edu.cn

**Aixin Yu,** yuaixin809@163.com

***Corresponding author, Tel: +86-10-6739-1726; Fax: +86-10-6739-1726; Municipal Engineering Department, College of Architecture and Civil Engineering, Faculty of Urban Construction, Beijing University of Technology, No.100 Xi Da Wang Road, Chao Yang District, Beijing 100124, P. R. China. E-mail:** liuyuankun@bjut.edu.cn **（Y. Liu）**


**Supplementary materials include 3 supplementary texts, 4 supplementary figures and 3 supplementary tables**

**Supplementary text**

**Text S1 List of chemical reagents**

**Text S2 Characterization**

**Text S3 Analytic methods**

**Supplementary figures**

**Figure S1. Zeta potential of MBC.**

**Figure S2. Effects of inorganic anions: (a) $Cl^-$, (b) $NO_3^-$, (c) $HCO_3^-$, (d) $H_2PO_4^-$, (e) Comparison of four inorganic ions; (f) Effect of HA; (g) CIP removal in actual water (Reaction conditions: pH, 6.4; initial CIP, 10 mg/L; MBC dosage, 0.6g/L; $H_2O_2$, 10 mM; temperature, 25 °C).**

**Figure S3. Mass spectrogram in the MBC/$H_2O_2$ condition.**

**Figure S4. The optimized conformation of CIP with atomic labeling.**

**Supplementary table**

**Table S1 Characteristics of the water samples.**

**Table S2 Degraded products of CIP by catalytic hydrogen peroxide decomposition.**

**Table S3 The atomic natural charge and bond length of CIP molecule.**

## Supplementary text

### Text S1 List of chemical reagents

Ciprofloxacin (CIP，$C_{17}H_{18}FN_3O_3$, 98%) was bought from Energy Chemical. Ferrous sulfate heptahydrate ($FeSO_4 \cdot 7H_2O$, ≥99.0%), copper sulfate pentahydrate ($CuSO_4 \cdot 5H_2O$, ≥99.0%), sodium oxalate ($Na_2C_2O_4$, ≥99.8%), tert butyl alcohol (TBA), potassium iodide (KI) were purchased from Aladdin Biochemical Corporation. Hydrochloric acid (HCl), sodium hydroxide (NaOH) were bought from Sinopharm.

### Text S2 Characterization

The surface characteristics and morphology of BC and MBC were studied by scanning electronic microscopy with energy dispersive X-ray spectroscopy (SEM, SU8020, Japan). The Brunauer-Emmett-Teller (BET, Micromeritics ASAP 2460, USA) was used to measure the specific surface areas ($S_{BET}$) and average pore diameters of BC and MBC, respectively. The compositions phases of FeCu, MBC and used MBC were detected by X-ray diffraction (XRD, BRUCKER D8 Advance, Germany) in 2θ from 5° to 90°. And the structural information of BC, MBC and used MBC was identified by Fourier transform infrared spectroscopy (FTIR, Nicolet 6700) within wavenumber range of 4000-400 $cm^{-1}$. The X-ray electron spectroscopy (XPS, Thermo ESCALAB 250 Xi) was used for examining the change of the chemical state of surface elements between fresh and used MBC. The BKT-4500 vibration sample magnetometer was used to VSM analysis of fresh and used MBC. The zeta potentials of MBC under different pH were obtained by using Zeta potentiometer (Malvern, UK).

**Text S3 Analytic methods**

The absorbance of the solution was detected at 277 nm by the UV-Vis spectrophotometer (UV-1900, Shimadzu, Japan). The total organic carbon (TOC) content was measured by a Multi TOC/TN Analyzer (vario TOC, Germany). The amount of leached metal ions was measured by using a ZA3300 atomic absorption spectroscopy (AAS, Hitachi, Japan). The contribution of ·OH in the reaction system was determined by Electron paramagnetic resonance (EPR, BRUCKER A300-10/12, Germany). The intermediates of CIP degradation were identified by HPLC-MS (Agilent 1290 Infinity/6460 LC/QQQ MS). $F^-$ and $NO_3^-$ were measured by ion chromatograph (883 Basic IC plus). The molecular structure of CIP was optimized by Density Functional Theory (DFT) at B3LYP/6-31+G* level via Gaussian 09.

## Supplementary figures

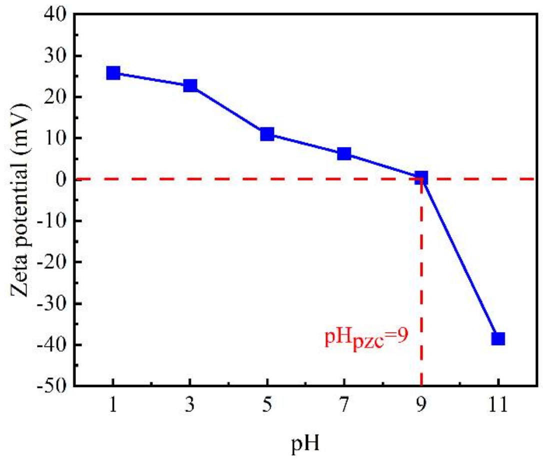


**FigureS1.** Zeta potential of MBC.

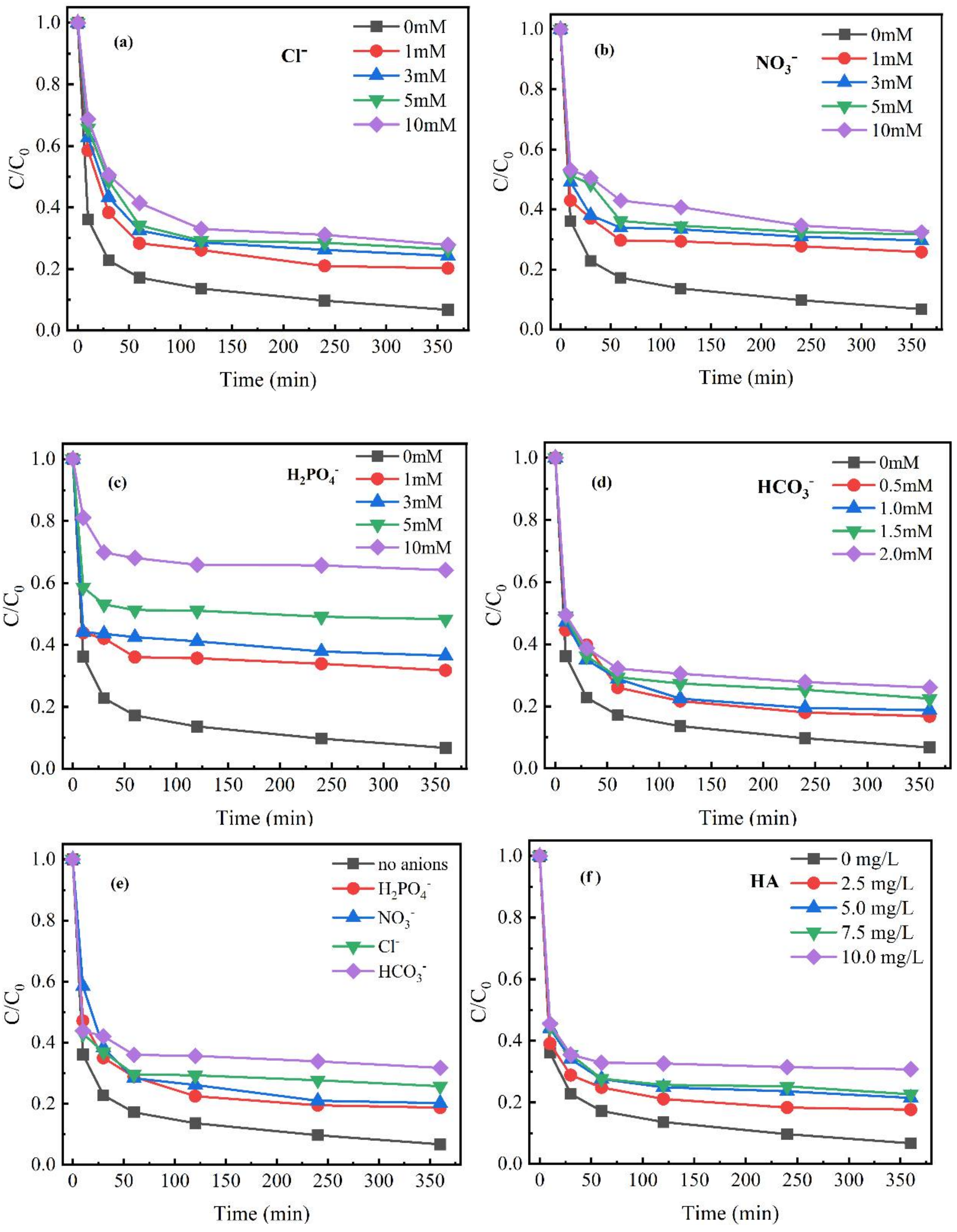
(a)
Cl-
0mM
1mM
3mM
5mM
10mM
C/C0
Time (min)
(b)
NO3-
0mM
1mM
3mM
5mM
10mM
C/C0
Time (min)
(c)
H2PO4-
0mM
1mM
3mM
5mM
10mM
C/C0
Time (min)
(d)
HCO3-
0mM
0.5mM
1.0mM
1.5mM
2.0mM
C/C0
Time (min)
(e)
no anions
H2PO4-
NO3-
Cl-
HCO3-
C/C0
Time (min)
(f)
HA
0 mg/L
2.5 mg/L
5.0 mg/L
7.5 mg/L
10.0 mg/L
C/C0
Time (min)

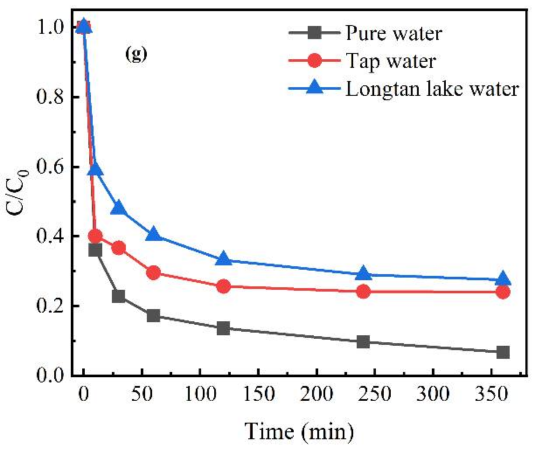


**Figure S2.** Effects of inorganic anions: (a) $Cl^-$, (b) $NO_3^-$, (c) $HCO_3^-$, (d) $H_2PO_4^-$, (e) Comparison of four inorganic ions; (f) Effect of HA; (g) CIP degradation in actual water (Reaction conditions: pH, 6.4; initial CIP, 10 mg/L; MBC dosage, 0.6g/L; $H_2O_2$, 10 mM; temperature, 25 °C).

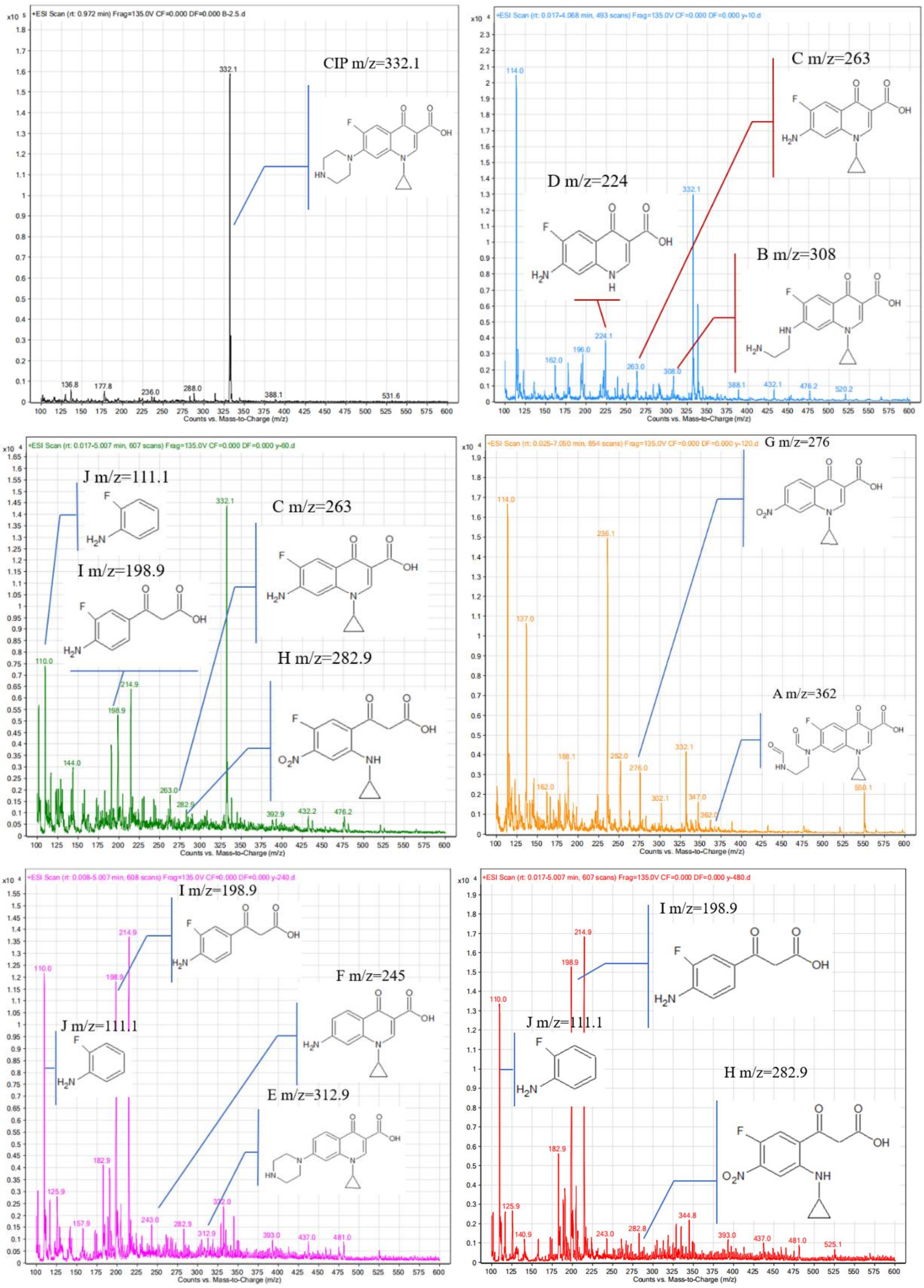


**Figure S3.** Mass spectrogram in the MBC/$H_2O_2$ condition.

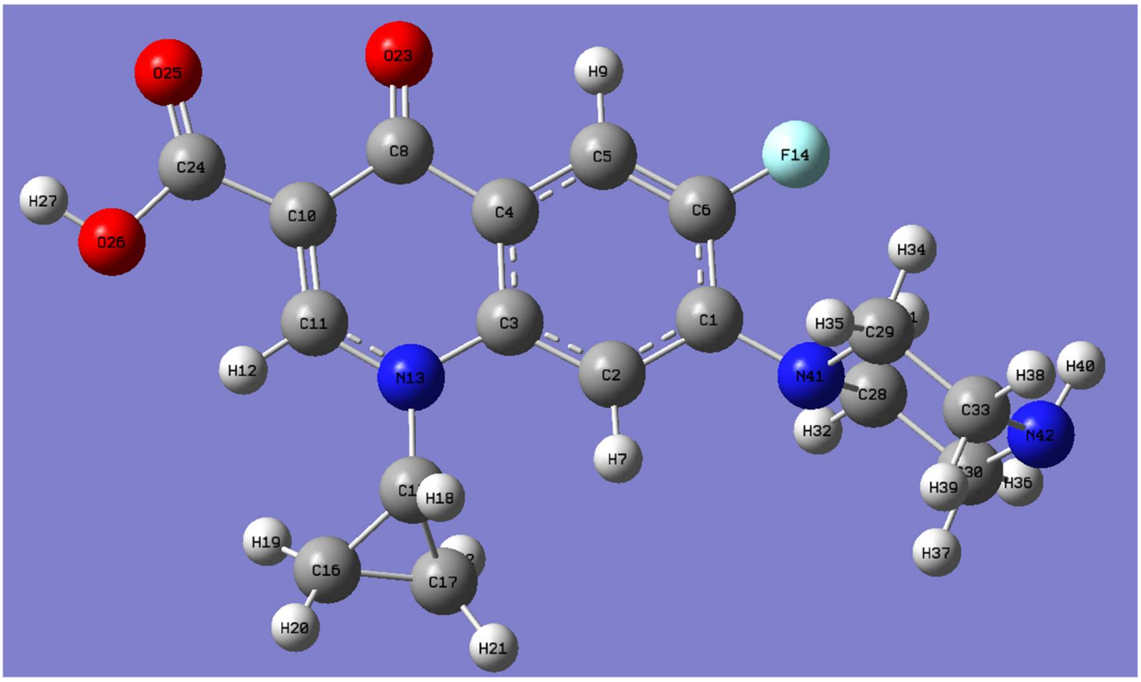


**Figure S4.** The optimized conformation of CIP with atomic labeling.

**Supplementary table**

**Table S1**

Characteristics of the water samples.

| Water samples | Water sample characteristics | | | |
|---|---|---|---|---|
| | Turbidity(NTU) | $UV_{254}$($cm^{-1}$) | DOC(mg/L) | pH |
| Tap water | 2.8 | 0.106 | 2.08 | 7.2 |
| Lake water | 9.5 | 0.172 | 5.44 | 8.0 |

**Table S2**
Degraded products of CIP by catalytic hydrogen peroxide decomposition.

| Name of the products | Molecular formula | m/z | Structural formula |
|---|---|---|---|
| CIP | $C_{17}H_{18}FN_3O_3$ | 332.1 | 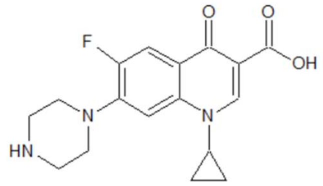 |
| A1 | $C_{17}H_{16}FN_3O_5$ | 362 | 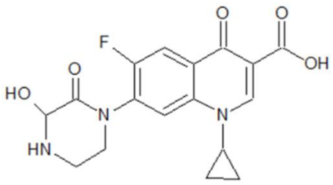 |
| A2 | | | 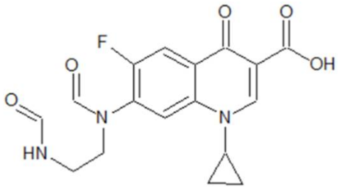 |
| B | $C_{15}H_{16}FN_3O_3$ | 308 | 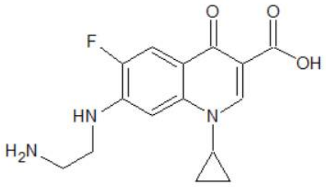 |
| C | $C_{13}H_{11}FN_2O_3$ | 263 | 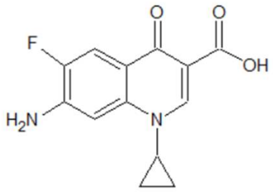 |
| D | $C_{10}H_7FN_2O_3$ | 224 | 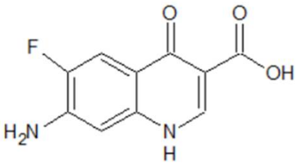 |
| E | $C_{17}H_{19}N_3O_3$ | 312.9 | 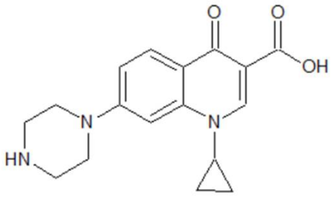 |

| | | | |
|---|---|---|---|
| F | $C_{13}H_{12}N_2O_3$ | 245 | 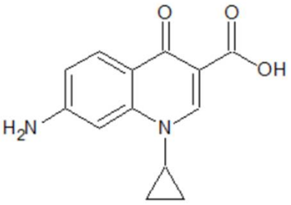 |
| G | $C_{13}H_{10}N_2O_5$ | 276 | 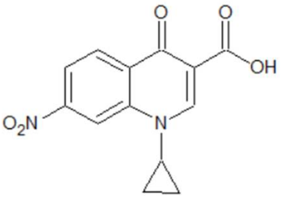 |
| H | $C_{12}H_{11}FN_2O_5$ | 282.9 | 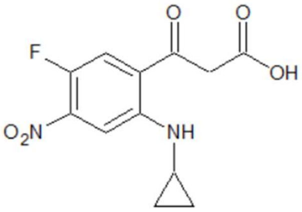 |
| I | $C_9H_8FNO_3$ | 198.9 | 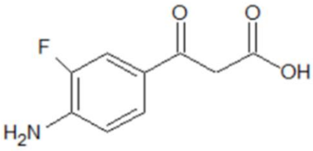 |
| J | $C_6H_6FN$ | 111.1 | 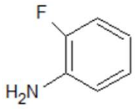 |

**Table S3**

The atomic natural charge and bond length of CIP molecule.

| Atom | Atomic natural charge | bond | Bond length |
|---|---|---|---|
| 1C | 0.202 | C(2)-C(1) | 1.392 |
| 2C | -0.219 | C(3)-C(2) | 1.405 |
| 3C | 0.319 | C(4)-C(3) | 1.410 |
| 4C | 0.034 | C(5)-C(4) | 1.402 |
| 5C | -0.277 | C(6)-C(5) | 1.378 |
| 6C | 0.374 | C(6)-C(1) | 1.409 |
| 8C | 0.393 | C(8)-C(4) | 1.493 |
| 10C | -0.064 | C(10)-C(8) | 1.470 |
| 11C | 0.040 | C(11)-C(10) | 1.373 |
| 13N | -0.541 | N(13)-C(11) | 1.358 |
| 14F | -0.293 | F(14)-C(6) | 1.354 |
| 15C | -0.002 | C(15)-N(13) | 1.450 |
| 16C | -0.334 | C(16)-C(15) | 1.506 |
| 17C | -0.305 | C(17)-C(16) | 1.505 |
| 23O | -0.491 | O(23)-C(8) | 1.227 |
| 24C | 0.557 | C(24)-C(10) | 1.478 |
| 25O | -0.448 | O(25)-C(24) | 1.210 |
| 26O | -0.614 | O(26)-C(24) | 1.375 |
| 28C | -0.133 | C(28)-N(41) | 1.474 |
| 29C | -0.134 | C(29)-N(41) | 1.474 |

| | | | |
|---|---|---|---|
| 30C | -0.151 | C(30)-C(28) | 1.535 |
| 33C | -0.151 | C(33)-C(29) | 1.534 |
| 41N | -0.488 | N(41)-C(1) | 1.429 |
| 42N | -0.528 | N(42)-C(30) | 1.466 |
| | | N(42)-C(33) | 1.466 |
| | | N(41)-C(29) | 1.474 |
| | | N(41)-C(28) | 1.474 |
| | | N(13)-C(3) | 1.407 |
